# Does this acute myocardial infarction patient have 9 lives as cats?: A case report and literature review

**DOI:** 10.1097/MD.0000000000044625

**Published:** 2025-09-19

**Authors:** Shuai Yang, Jing Sun, Hu Zhai, Li Miao, Huishuai Dong, Hongshuo Jiao, Gang Wang

**Affiliations:** a Department of Heart Center, The Third Central Hospital of Tianjin, Tianjin Key Laboratory of Extracorporeal Life Support for Critical Diseases, Artificial Cell Engineering Technology Research Center, Tianjin, China; b Department of Cardiology, Characteristic Medical Center of Chinese People’s Armed Police Force, Tianjin, China.

**Keywords:** acute myocardial infarction, coronary angiogram, intravascular ultrasound, very late stent thrombosis

## Abstract

**Rationale:**

Very late stent thrombosis (VLST) is associated with high mortality rates. The use of endoluminal imaging to identify the causes of VLST is crucial. Here, we report a case of VLST occurring 7 times over 14 years, wherein both stent fracture and malapposition were confirmed by endoluminal imaging.

**Patient concerns:**

A 66-year-old male patient had experienced sudden, recurrent myocardial infarctions 7 times over a period of 14 years, receiving 5 stents and 2 drug-coated balloons. An Electrocardiogram showed stent thrombosis-segment elevation in the inferior wall leads. Emergency coronary angiography demonstrated total occlusion of the proximal right coronary artery. Anticoagulation, thrombus aspiration and intracoronary thrombolysis were performed to treat the coronary thrombosis.

**Diagnoses:**

The etiology of VLST was confirmed as stent fracture and malapposition, based on endoluminal imaging.

**Interventions:**

Stent implantation was performed following balloon angioplasty using a non-compliant balloon.

**Outcomes:**

The patient remained asymptomatic and free of adverse cardiovascular events during an 18-month follow-up.

**Lessons:**

“Three-step” strategy is suggested for VLST management. Therefore, endoluminal imaging is important.

## 1. Introduction

Stent thrombosis (ST) is a rare yet highly fatal complication, often associated with patient-, lesion-, procedure-, or drug-related factors.^[1]^ Very late ST (VLST) typically manifests as unstable angina pectoris, acute myocardial infarction (AMI), or sudden death occurring more than 1 year post-stent implantation, with thrombus presence confirmed either at autopsy or through coronary angiogram (CAG). Early ST is typically procedure-related, The HORIZON-AMI trial showed that a stent area < 5.0mm^2^, severe residual stenosis, stent edge dissection, stent underexpansion and obvious tissue prolapse are all critical factors for thrombosis,^[2]^ In contrast, late ST or VLST involves delayed endothelialization, neoatherosclerosis, malapposition and stent fracture.^[3]^ Therefore, the ESC and ACC/AHA guidelines recommend using endoluminal imaging, such as intravascular ultrasound (IVUS) or optical coherence tomography (OCT), to guide and optimize percutaneous coronary intervention.^[4–6]^ Here, we present the case of a patient with AMI from VLST over a span of 14 years due to stent fracture and malapposition, as confirmed by IVUS.

## 2. Case report

**Figure 1.**
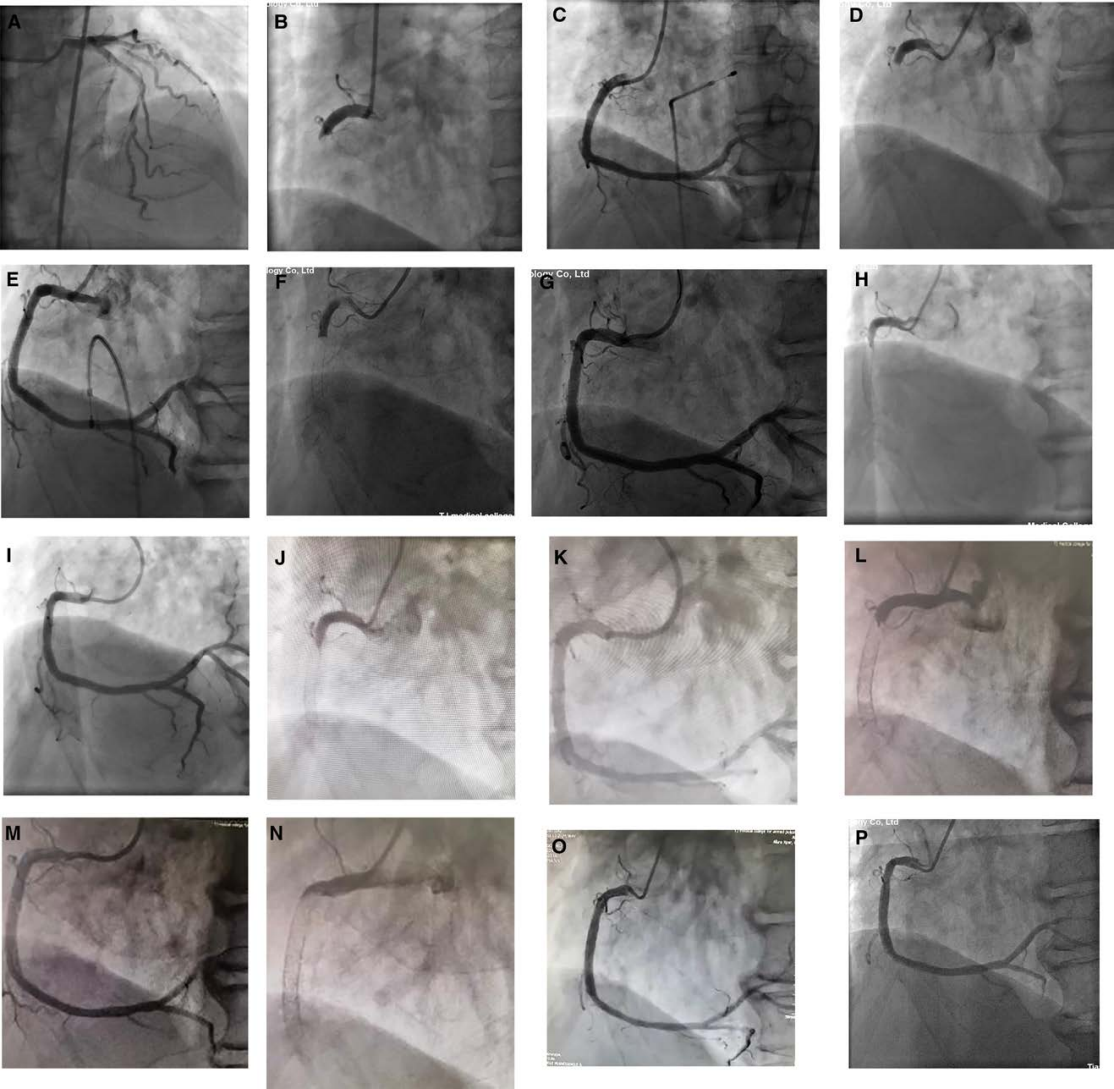
(A) Mild sclerosis of the LAD and LCX. (B) Complete occlusion of the mid-RCA. (C) Stent in RCA (3.5/18 EXCEL, 4.0/29 partner). (D) 1st VLST (3 yr later). (E) Stent (3.5/18 Xience prime). (F) 2nd VLST (six years later). (G) Stent (4.0/30 resolute). (H) 3rd VLST (8 yr later). (I) Balloon dilation and antithrombotic therapy. (J) 4th VLST (9 yr later). (K) Stent (4.0/33 Firebird2). (L) 5th VLST (11 yr later). (M) Drug-coated balloon (3.5/30 restore). (N) 6th VLST (12 yr later). (O) Drug-coated balloon (3.5/30 restore). (P) CAG showed good results with mild hyperplasia in the stent (13 yr later). CAG = coronary angiogram, LAD = left anterior descending, LCX = left circumflex, RCA = right coronary artery, VLST = very late stent thrombosis.

**Figure 2.**
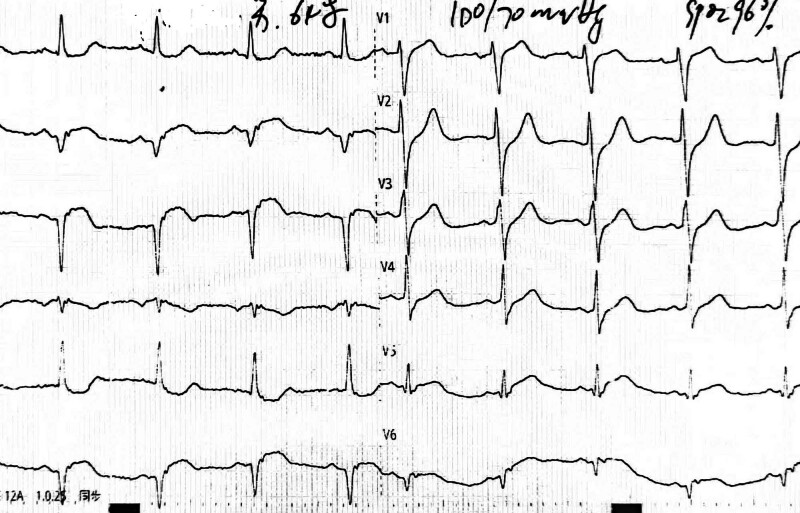
The ECG revealed a sinus heart rate at 70 beats per minute, with a pathological Q wave and ST-segment elevation of 0.2–0.3 mv in leads II, III, and aVF. ECG = electrocardiogram.

**Figure 3.**
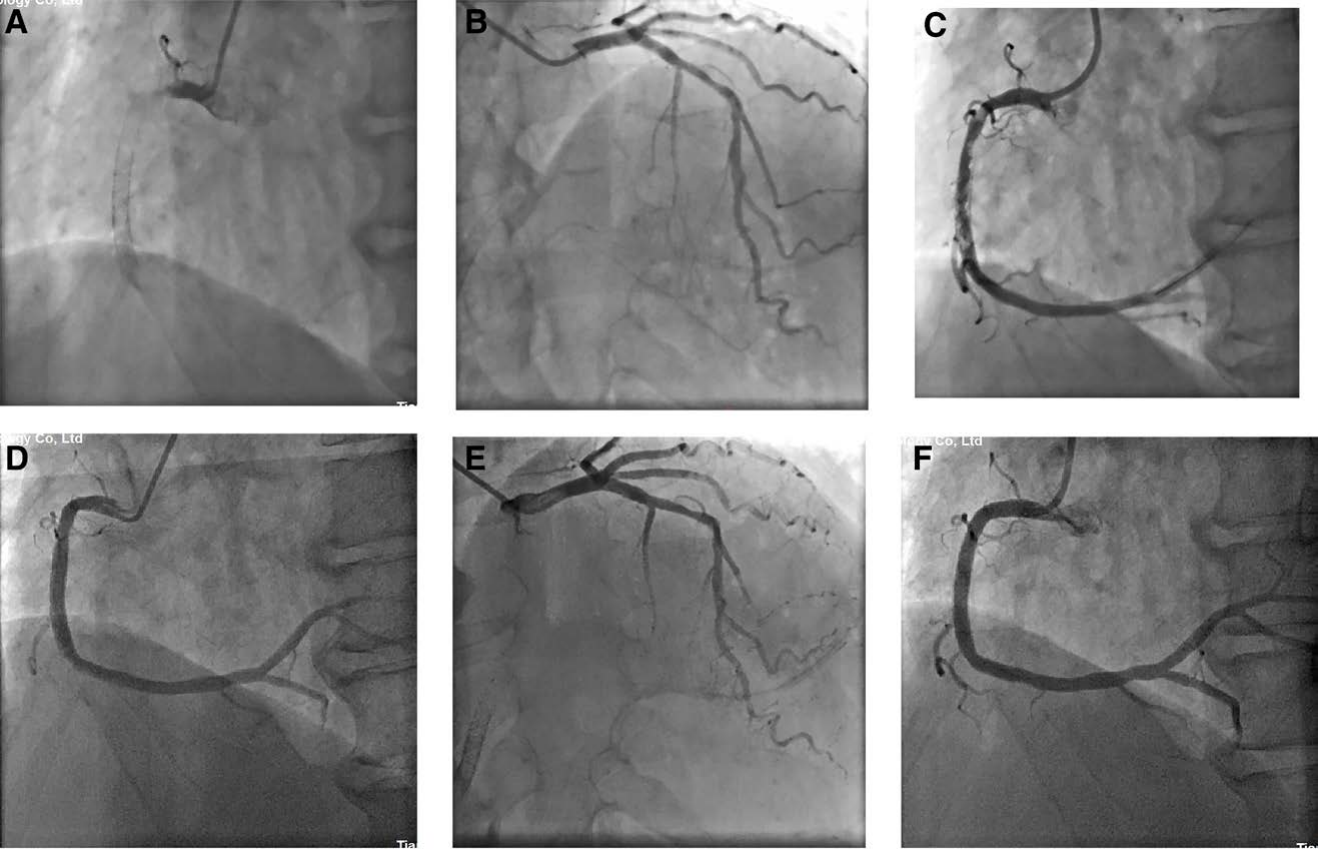
(A) Seventh VLST in RCA. (B) Mild stenosis in the proximal segment of LAD and LCX. (C) Coronary artery thrombolysis (rhTNK-tPA) with a large amount of thrombus. (D) Thrombus disappeared with mild plaque in the proximal segment of RCA (18 days later). (E) Mild stenosis in the proximal segment of LAD and LCX. (F) 4.0/24 stent (Partner) showed good results with no residual stenosis in the RCA stent. LAD = left anterior descending, LCX = left circumflex, RCA = right coronary artery, VLST = very late stent thrombosis.

**Figure 4.**
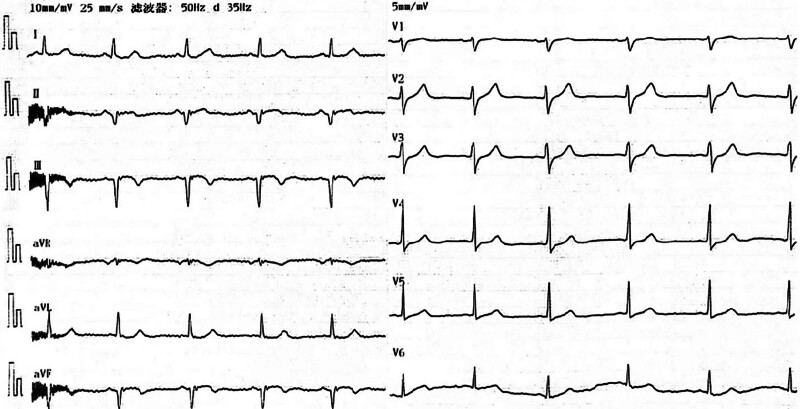
The ECG revealed a sinus heart rate at 70 beats per min, with a pathological Q wave and elevated ST-segment resolution in leads II, III, and aVF. ECG = electrocardiogram.

A 66-year-old male patient was admitted to the Heart Center with chest pain persisting for 14 years, he had been experiencing “recurrent episode, with the latest occurring the day before admission.” The patient had been diagnosed with AMI of the inferior wall at a local hospital 14 years earlier. A coronary angiogram (CAG) revealed total occlusion of the proximal segment of the right coronary artery (RCA) and mild stenosis in the left anterior descending (LAD) and left circumflex (LCX) arteries (Fig. 1A and B). Thrombus aspiration and stent implantation (3.5 × 18 mm Excel, 4.0 × 29 mm partner) were performed in the RCA (Fig. 1C). Over the past 14 years, the patient had experienced 6 recurrent episodes of AMI of the inferior wall. Emergency CAG confirmed VLST in the proximal to mid-segment of the RCA every time. Thrombus aspiration and implantations of 5 second-generation drug-eluting stents (DES) and 2 drug-coated balloons were performed in the proximal to mid-segment of the RCA (Table 1, Fig. 1D–O). After discharge, the patient was treated with oral aspirin, clopidogrel sulfate (1 year), and atorvastatin. Three months before this admission, a repeat CAG revealed no significant in-stent restenosis (Fig. 1P). On admission, blood pressure was 100/70 mm Hg (1 mm Hg = 0.133 kPa), and heart rate was 70 beats/min. An eletrocardiogram (ECG) revealed pathological Q waves with ST-segment elevation 0.2 to 0.3mV in leads II, III, and aVF (Fig. 2). The troponin I level was higher than normal at 5.85 ng/mL (reference: <1.68 ng/mL), and the creatine kinase-MB was 41U/L (reference: 0–25U/L). Echocardiography revealed a left ventricular ejection fraction (LVEF) of 55% with hypodynamic motion in the inferior wall. The patient was diagnosed with acute inferior myocardial reinfarction and hyperlipidemia. After administering 600 mg of clopidogrel sulfate, an emergency CAG was performed. The CAG revealed total occlusion in the proximal stent of the RCA, with mild atherosclerosis in the LAD and LCX arteries (Fig. 3A and B). Thrombus aspiration and intracoronary thrombolysis were performed using a recombinant human TNK tissue plasminogen activator (rhTNK-tPA 8mg, Shijiazhuang Pharmaceutical, China), however, a significant thrombus burden persisted in the stent (Fig. 3C). The patient reported relief from chest discomfort. The optimized drug therapy was continued postoperatively. ECG showed significant ST-segment resolution in leads II, III, and aVF (Fig. 4).

**Table 1:** PCI history in past 14 years.

MI time	RCA	Aspiration	IC thrombolysis	Stent/balloon	DCB
2010	Proximal and mid-segment	Yes	No	3.5/18 EXCEL, 4.0/29 Partner	No
2013	Proximal and mid-segment	Yes	rt-PA 10 mg	3.5/18, Xience Prime	No
2016	Proximal and mid-segment	Yes	No	4.0/30 Resolute	No
2018	Proximal and mid-segment	Yes	No	Balloon inflate	No
2019	Proximal and mid segment	Yes	No	4.0/33 Firebird	No
2021	Proximal	Yes	No	No	3.5/30 restore
2022	Proximal	Yes	No	No	3.5/30 restore

DCB = drug-coated balloon, IC = intra coronary, MI = myocardial infarction, RCA = right coronary artery, rt-PA = alteplase.

Dual antiplatelet therapy (DAPT, aspirin 100 mg and clopidogrel 75 mg once daily) was continued during hospitalization. Platelet aggregation via the arachidonic acid and adenosine diphosphate pathways was accessed using thromboelastography, revealing aggregation rates of 8% (reference range: 56%–82%) and 9% (reference range: 53%–87%), respectively. Platelet inhibition was adequate, ruling out antiplatelet resistance as a cause of ST. Thrombophilia was excluded as AT-III, protein C, and protein S were within normal range. Anticardiolipin antibody test result negative.

**Figure 5.**
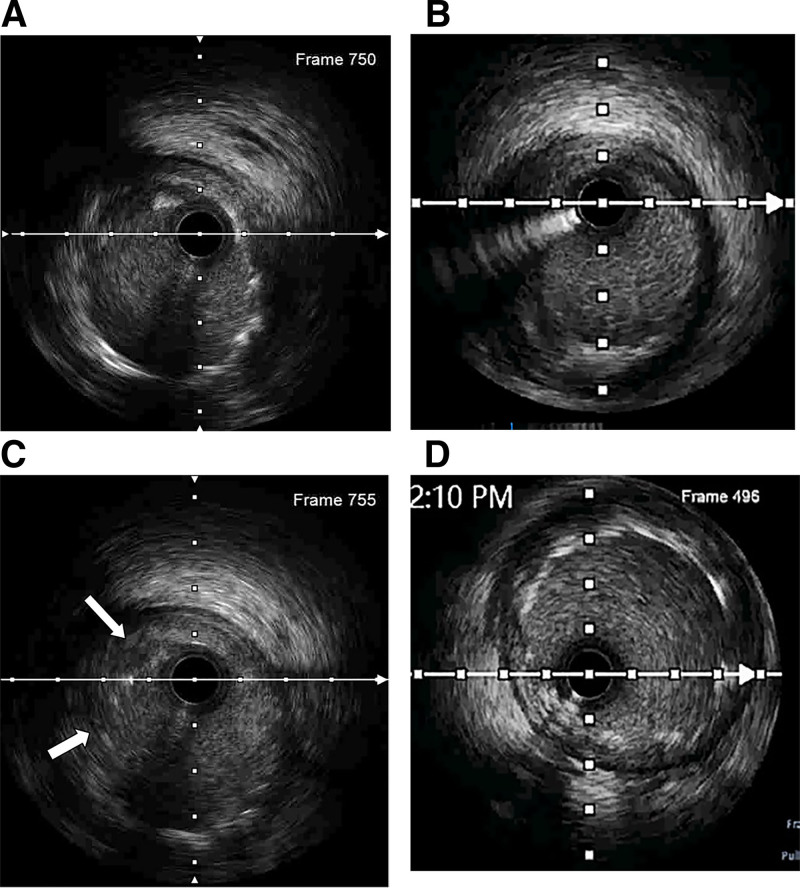
(A) Multi-layer stents in mid-segment of RCA. (B) local absence of metallic stent struts. (C) Stent malapposition from 8 to 11 o’clock direction (white arrow). (D) Stent expansion and good apposition. RCA = right coronary artery.

Eighteen days later, CAG revealed resolution of RCA ST and mild atherosclerosis in the proximal RCA and LAD and LCX arteries (Fig. 3D and E). Given the recurrence of the VLST, IVUS was performed. IVUS revealed multiple layers of stent struts in the mid-segment of the RCA (Fig. 5A) and malapposition and fracture in the proximal-mid-segment of the RCA (Fig. 5B and C). A 4.0 × 12 mm non-compliant balloon (Aeronaut) was predilated, followed by the implantation of a 4.5 × 24 mm stent (GuReater, Lepu Medical). A 4.5 × 8mm non-compliant balloon (Abbott) was post-dilated to achieve good coronary imaging (Fig. 3F). IVUS demonstrated adequate stent expansion, absence of stent malapposition and fracture with a minimal lumen area of 15.9mm² after the operation (Fig. 5D). Ezetimibe was added to lower the low-density lipoprotein cholesterol (2.47mmol/L). The patient reported no chest tightness during the 18-month follow-up period. Due to the presence of multiple stent layers without endothelialization, lifelong DAPT was recommended.

## 3. Discussion

ST is defined as the thrombotic occlusion of a coronary artery stent. Patients with ST usually present as unstable angina pectoris, AMI, or sudden death.^[6]^ According to the Academic Research Consortium criteria, ST can be classified based on the stent type (bare-metal ST, first-generation drug-eluting ST, or second-generation drug-eluting ST), event timing (early ST [within 1 month], late ST [1 to 12 months], or VLST [after 12 months]), and event certainty (definite, probable, or possible ST).^[7]^ It is often associated with patient-, lesion-, procedure-, and drug-related factors. Patient factors included clinical conditions (smoking, chronic renal disease, diabetes mellitus, low LVEF) and lesion factors (multiply lesions, calcification, long or total occlusion lesions, bifurcation, or thrombus-containing lesion). Procedural factors include TIMI flow grade < 3, residual stenosis or dissection, implantation of additional stents, stent delayed endothelialization, neoatherosclerosis, and stent malapposition.^[5]^ Sohail found that patients with VLST were more likely to be male, of White ethnicity, have previous MI, diabetes mellitus, low LVEF, hypercholesterolemia, hypertension, cardiogenic shock, more and longer stents use, and more thrombus aspiration catheter.^[8]^ Enomoto studied 595 patients with definite ST in second-generation DES, of whom 29 patients experienced VLST during 31 months of follow-up. The incidence of VLST was 4.8%.^[9]^ Choi S-Y demonstrated that IVUS or OCT can help in identifying mechanical factors such as stent under-expansion, in-stent tissue protrusion, residual stenosis, and edge dissection that contribute to decreasing ST risk.^[2]^ Ke Nan Huang observed more DAPT discontinuation in patients with second-generation DES than in those with first-generation DES in VLST.^[10]^ Taniwaki examined 64 patients with VLST using OCT and found malapposition, neoatherosclerosis, uncovered struts, and underexpansion as the main etiologies, in that order. Longitudinal extension of malapposed (34.5%), neoatherosclerosis (27.6%), and uncovered stents (12.1%) were the three important factors of thrombus formation in VLST. Late-acquired malapposition (positive remodeling) or persistent malapposition requires further research.^[11]^ Gomez-Lara J found that stent under-expansion was the most frequent finding in patients with VLST undergoing IVUS-guided percutaneous coronary intervention (PCI) (>75%), suggesting the use of a larger balloon to correct malapposition by expanding stent sizes.^[12]^ In the current case, the underlying mechanism of repeated VLST may have involved the following factors: stent malapposition and fracture, delayed endothelialization of the multiple stent layers with drug overload, chronic inflammation, impaired vascular healing promoting fibrin and platelet deposition, and platelet activation due to exposed fractured stent struts.

The 2018 European consensus recommends the use of IVUS/OCT to evaluate ST mechanisms. We emphasize the following three critical aspects: For malapposition, appropriately sized balloon expansion is advised; however, for stent fracture or plaque rupture, a new stent may be necessary; Intensified antiplatelet therapy in case of poor endothelialization; and Platelet function and genotype testing should be routinely performed to optimize antiplatelet therapy.

## 4. Conclusion

Base on our case, we propose a “three-step” strategy for VLST management: acute thrombus reduction with aspiration and/or low-dose intracoronary thrombolysis. identification of underlying causes (malapposition, delayed neoatherosclerosis, fracture) using endoluminal imaging after thrombus clearance; and targeted mechanical and medical treatment with comprehensive management. Staged endoluminal imaging after thrombus clearance is essential to guide optimal treatment strategies for patients with VLST.

## Author contributions

**Data curation:** Shuai Yang, Jing Sun, Gang Wang.

**Investigation:** Hu Zhai, Li Miao.

**Methodology:** Huishuai Dong, Hongshuo Jiao.

**Writing – original draft:** Shuai Yang.

**Writing – review & editing:** Gang Wang.
